# Culinary Nutrition Education Programs in Community-Dwelling Older Adults: A Scoping Review

**DOI:** 10.1007/s12603-022-1876-7

**Published:** 2023-01-24

**Authors:** Maryam M. Alghamdi, T. Burrows, B. Barclay, S. Baines, C. Chojenta

**Affiliations:** 1 School of Health Sciences, College of Health, Medicine and Wellbeing, University of Newcastle, Callaghan, NSW 2308 Australia; 2 Clinical Nutrition Department, College of Applied Medical Sciences, Taibah University, Madinah, Saudi Arabia; 3 Food and Nutrition program Hunter Medical Research Institute, Callaghan, NSW 2308 Australia; 4 Centre for Active Living and Learning, School of Education, College of Human and Social Futures, University of Newcastle, Callaghan, NSW 2308 Australia; 5 School of Medicine and Public Health, College of Health, Medicine and Wellbeing, University of Newcastle, Callaghan, NSW 2308 Australia; 6 Hunter Medical Research Institute, New Lambton Heights, NSW 2305 Australia

**Keywords:** Nutrition education, culinary, cooking, review, ageing

## Abstract

**Background:**

Culinary nutrition education programs are increasingly used as a public health intervention for older adults. These programs often integrate nutrition education in addition to interactive cooking workshops or displays to create programs suitable for older adults’ needs, ability and behaviour change. Synthesising the existing literature on nutrition education and interactive cooking programs for older adults is important to guide future program development to support healthy ageing.

**Objectives:**

To determine the extent of published literature and report the characteristics and outcomes of interactive culinary nutrition education programs for older adults (> 51 years).

**Design:**

This scoping review followed the PRISMA-ScR guidelines recommended for reporting and conducting a scoping review.

**Methods:**

Five databases were searched of relevant papers published to May 2022 using a structured search strategy. Inclusion criteria included: older adults (≥ 51 years), intervention had both an interactive culinary element and nutrition education and reported dietary outcome. Titles and abstracts were screened by two reviewers, followed by full-text retrieval. Data were charted regarding the characteristics of the program and outcomes assessed.

**Results:**

A total of 39 articles met the full inclusion criteria. The majority of these studies (n= 23) were inclusive of a range of age groups where older adults were the majority but did not target older adults exclusively. There were large variations in the design of the programs such as the number of classes (1 to 20), duration of programs (2 weeks to 2 years), session topics, and whether a theoretical model was used or not and which model. All programs were face-to-face (n= 39) with only two programs including alternatives or additional delivery approaches beside face-to-face settings. The most common outcomes assessed were dietary behaviour, dietary intake and anthropometrics.

**Conclusion:**

Culinary nutrition education programs provide an environment to improve dietary habits and health literacy of older adults. However, our review found that only a small number of programs were intentionally designed for older adults. This review provides a summary to inform researchers and policy makers on current culinary nutrition education programs for older adults. It also recommends providing face-to-face alternatives that will be accessible to a wider group of older adults with fewer restrictions.

**Electronic Supplementary Material:**

Supplementary material is available for this article at 10.1007/s12603-022-1876-7 and is accessible for authorized users.

## Introduction

**P**oor nutrition is a key contributor to the global burden of disease (1). It increases the risk of developing non-communicable diseases and mortality (1). A high-quality diet that is characterised by variety and that is high in vegetables, fruits and whole grains is well recognised to improve quality of life, better manage chronic diseases and to reduce mortality (2, 3). While nutrition is important at all ages, it is of particular importance for older adults (aged ≥ 65 years) and is fundamental to promoting functional ability that enables wellbeing in older age (4).

A number of physiological, psychosocial, and personal factors play key roles in older adults’ food choices (5). Age-related physiological changes can include changes in appetite, taste and smell, oral health and the efficiency of chewing (5, 6). A large and growing body of literature has reported an established connection between these physiological changes and sub-optimal nutritional intake in older adults (7). Changes in taste and smell affect food intake by reducing the enjoyment of eating and appetite, which in turn can affect portion sizes and food choices (6, 8). The loss of taste can lead to higher consumption of food high in salt and sugar to increase meal palatability (5, 6). In addition, poor dentition and compromised chewing tends to increase with age, and this may result in changes to food choices (5), for example, reducing the intake of certain foods that are crunchy, dry, solid and harder to chew such as nuts, fruit, vegetables and meats.

Psychosocial factors such as living alone, and bereavement and grief following the loss of a partner have also been reported to affect older people’s food intake. The number of older adults who live alone is about 28% in the United States, while in Australia, approximately about 25% of people aged 65 and over live alone (9). Living alone is associated with low motivation for older adults to shop, prepare and eat meals. Research suggests that people living alone eat less diverse food and lacks some core food groups such as fish, fruit and vegetables compared to people not living alone (10). They also skip meals or replace them with snacks (11, 12). This can occur as a result of losing pleasure while eating without companionship, which appears to affect women more than men (11, 13). Furthermore, personal factors such as nutrition knowledge, cooking skills and income affect food intake (14). Food budget directly impacts food choice and may lead to poor dietary intake, which may decrease with age and retirement (15). Cooking skills in older people varies based on education level and gender with women having a higher level of cooking skills than men in many populations (12, 16–18). While having good cooking skills does not necessarily ensure that older people meet their energy requirement, people with better cooking skills have shown to have a healthier diet intake compared with older people with lower level skills (12, 18)

Nutrition education programs and culinary classes are increasingly used as a public health intervention (19, 20). Combining nutrition education and culinary classes in a program helps individuals to gain understanding on how to replicate healthy meals and focus on healthy nutrition patterns (19). This integration allows participants to actively engage in the program, enhancing autonomy, empowerment, and behaviour modification (21). These are often referred to in the literature as culinary nutrition (22). The level of education and amount of cooking within existing programs is highly variable and can include nutrition information, group-based hands-on activities such as food demonstrations and taste-testing, as well as discussions on lifestyle topics such as meal planning, food safety and a supermarket tour (23). Integrating a hands-on cooking experience is important to provide cooking literacy and has shown to increase the consumption of fruit and vegetables (24). Depending on the style of program delivery and if the program is group-based these programs allow participants to meet new people and expand their social networks (21). Programs based on behavioural theory are more likely to achieve the desired change as they focus on activities that help individuals to reflect upon their risk behaviours and help change them to healthier habits, specifically addressing barriers and facilitators to eating (21, 25). Various theoretical frameworks have been used in the design of interventions based on the health problem, intervention goals, and practices (26). The most common theory applied to cooking programs is the social cognitive theory (27).

Research has been published to describe either nutrition education programs (20) or culinary education programs in children, women during childbearing years or adults (19, 28, 29). However, no review has described programs that combined these two modules together and in older adults. Existing culinary nutrition education programs are varied, with some specifically designed for older adults exclusively while others include older adults in their broader population. This scoping review synthesises the existing literature of culinary nutrition education programs for older adults aimed at improving the health and wellbeing of community-dwelling older adults in terms of the number, characteristics, strategies, delivery approach and theoretical framework used in these programs. This scoping review’s findings will help shape decisions regarding future evidence-based program development by identifying components of culinary nutrition education programs for older adults associated with success. This scoping review will answer the following questions: (1) What are the characteristics of culinary nutrition education programs for older adults? (2) What are the measured outcomes of culinary nutrition education interventions for older adults?

## Research design and Methods

This scoping review was guided by the Preferred Reporting Items for Systematic Reviews and Meta-Analyses for Scoping Review (PRISMA-ScR) guidelines and a study protocol (supplementary appendix A) that was developed prior to conducting the search (30).

### Literature search

Five databases (MEDLINE (Ovid), EMBASE (Ovid), CINAHL, Scopus and Cochrane Library) were searched in May 2022 for all previous entries with no date limits. Keywords included “Cooking/or cook*,” “culinary or eating or dietary,” “taste test* or food sampl*,” “((cooking or food or recipe* or meal*) adj3 demonstrat*),” “((“meal* or food) adj3 (budget* or plan* or skill* or knowledge or education* or prepar* or literac* or shop*)), AND “nutrition* adj5 (intervention* or program* or workshop or workshops or knowledge or education)),” AND “aged/,” “((aged or elderly or older or senior) adj3 (male* or female* or men or women or citizen* or person or people or population))”. The search strategy was pilot tested and further refined with the assistance of a senior librarian before the final search was conducted.

### Inclusion and exclusion criteria

Studies were eligible if they included older adults (≥ 51 years) and the intervention had both an interactive cooking element and nutrition education. Studies that were not exclusively for older people, but where some participants were aged ≥ 51 years were also included if the results were reported and stratified by age. This strategy was employed to provide a comprehensive synthesis of all culinary nutrition education programs whether they were specifically designed for older adults or for people of any age. An interactive cooking element was defined as any of the following: the cooking of meals, recipe demonstration, taste testing or food sampling. Participants needed to be living independently and be preparing their own meals. Studies were included regardless of comparator used, or methodological approach. Qualitative studies were included. Studies needed to report dietary outcomes which included but was not limited to food intake and dietary behaviours and be written in English. Studies were excluded if they did not combine nutrition education and interactive cooking activities (i.e. reported either one of these elements individually), were not in older adults (<51 years) or results were not stratified by age, did not measure dietary outcomes (e.g., measured physiological outcomes or presented an evaluation of program content only), not an original paper (e.g., review articles, commentaries, reports), if full text was not available (e.g., conference abstract), or if they were in a language other than English.

#### Screening

Studies were screened by two independent reviewers (MA and BB). Title and abstracts were first screened using Covidence (systematic review software). Remaining full text studies were extracted and screened for inclusion. A third reviewer (CC or TB) resolved any disagreements.

### Data extraction

Data extracted included study details (including study design, program activities, class frequency and duration, education topics), participant characteristics, program characteristics, and program outcome measures (including dietary intake, anthropometric measurements, health measures, knowledge, attitudes and behaviours). Data were synthesised descriptively to report characteristics of the program and outcomes. Data extraction was performed by one reviewer (MA) and validated by another (BB, TB or CC), with any discrepancies resolved through consensus. Dietary outcomes were categorised into major groupings such as overall food intake based, dietary behaviours, malnutrition, fruit and vegetables and nutrient/food based, all other variables were considered non dietary outcomes. As this was a scoping review, no quality appraisal was performed for this study.

## Results

**Figure 1:**
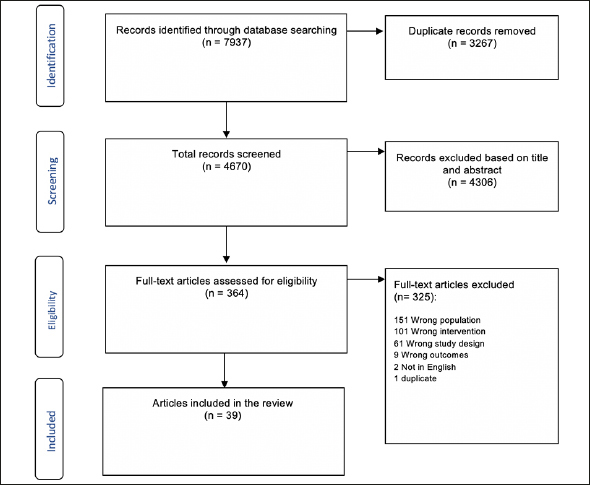
PRISMA flow diagram

In total, 7937 articles were identified by the search. After removing duplicates, 4670 were eligible for title and abstract screening. Of these, 364 were eligible for full-text screening and 39 articles met the inclusion criteria of this review (Figure 1). The major reasons for exclusion were; wrong population (<50 years) (n=151), wrong intervention (did not combine nutrition education and interactive cooking activities but reported either one of these elements individually) (n=101), or wrong study design (i.e. report, review articles, conference abstracts) (n=61).

### Overall characteristics of nutrition and cooking programs

Studies were conducted in 14 countries with the majority in the United States (n=24) (31–53) followed by Brazil (n=2). The majority (n=29) of the articles were published between 2011 and 2021 (32, 34–36, 38, 40–46, 49–51, 53–66). Study designs were primarily pre-post studies (n=20) (33–35, 38–40, 43, 44, 47–51, 55–57, 59, 60, 65, 67), followed by randomised control trial (n=15) (37, 41, 42, 45, 46, 52, 53, 58, 61–64, 66, 68, 69) and quasi-experimental studies (n=4) (31, 32, 36, 54). Program duration ranged from 2 weeks to 2 years, and the number of classes ranged from 1 to 20 classes (Table 1). A majority of the educators were reported to be a dietitian and/or nutritionist (n=17) (31, 32, 35, 42, 43, 47–51, 55, 57, 61–63, 67, 69), a registered nurse and/or trained educator (n=10) (33, 34, 37, 44, 45, 52, 53, 58, 60, 64). Thirteen studies did not report the qualifications of the class instructor (36, 38–41, 46, 54, 56, 58, 59, 65, 66, 68).

**Table 1:** Characteristics of included studies culinary nutrition education programs for older adults (N=39)

		**All programs n (%)**	**Targeted older adults n (%)**	**Targeted all adults but included older adults n (%)**
		**n=39**	**n=16**	**n=23**
Country	Australia	1 (2.5%)	-	1 (4.3%)
	Brazil	2 (5.3%)	-	2 (8.7%)
	Canada	1 (2.5%)	1 (6.2%)	-
	China	1 (2.5%)	1 (6.2%)	-
	Indonesia	1 (2.5%)	1 (6.2%)	-
	Ireland	1 (2.5%)	1 (6.2%)	-
	Italy	1 (2.5%)	-	1(4.3%)
	Malaysia	1 (2.5%)	1 (6.2%)	-
	Poland	1 (2.5%)	1 (6.2%)	-
	South Africa	1 (2.5%)	-	1 (4.3%)
	Sweden	1 (2.5%)	1 (6.2%)	-
	Taiwan	1 (2.5%)	1 (6.2%)	-
	Thailand	1 (2.5%)	1 (6.2%)	-
	United States	24 (61.5%)	7 (43.7%)	17 (74%)
	Vietnam	1 (2.5%)	-	1 (%6.2)
Participant inclusion criteria: Sex	No sex criteria	29 (74.3%)	15 (93.7%)	14 (61%)
	Female only	10 (25.6%)	1 (6.2%)	9 (39.1%)
Study design	Randomized controlled trial	14 (35.9%)	4 (25%)	9 (39.1%)
	Pre- & post	21 (53.8%)	9 (56.2%)	13 (56.5%)
	Quasi-experimental	3 (7.7%)	2 (12.5%)	1 (4.3%)
Sample size	< 50	15 (38.5%)	6 (37.5%)	9 (39.1%)
	51 – 100	10 (25.6%)	5 (31.2%)	5 (22%)
	101 – 500	7 (18%)	4 (25%)	3 (13%)
	501 – 1000	1 (5.3%)	-	1 (4.3%)
	1001 – 2200	3 (7.7%)	1 (6.2%)	3 (13%)
Stakeholder engagement		10 (25.6%)	4 (25%)	6 (26%)
Interactive components	Cooking	11 (28.2%)	2 (12.5%)	9 (39.1%)
	Eating/sharing meals	5 (12.8%)	2 (12.5%)	3 (13%)
	Food samples/taste testing	19 (48.7%)	6 (37.5%)	13 (56.5%)
	Food demonstrations	13 (33.3%)	5 (31.2%)	8 (34.7%)
	Videos	5 (12.8%)	1 (6.2%)	5 (22%)
	Online resources	2 (5.1%)	1 (6.2%)	1 (4.3%)
Program duration	1 to 4 weeks	5 (12.8%)	2 (%12.5)	3 (13%)
	5 to 12 weeks	10 (25.6%)	5 (31.2%)	5 (22%)
	13 to 25 weeks	7 (17.9%)	4 (%25)	3 (13%)
	26 to 51 weeks	5 (12.8%)	1 (6.2%)	4 (17.4%)
	52 to 104 weeks	8 (20.5%)	3 (18.7%)	5 (22%)
	Not reported	4 (10.2%)	1 (6.2%)	3 (13%)

### Program Participants

This review identified 16 culinary nutrition education programs that were specifically designed for older adults aged ≥ 51 years (35, 36, 39, 40, 51, 54–56, 58–60, 64, 66, 67, 69) and 23 that were not specifically designed for older adults but included them in a broader population group (31–34, 37, 38, 41–50, 52, 53, 57, 61–63, 65, 68). Sample size ranged from 8 to 2519 participants, with the majority having less than 100 participants. Of the 39 studies, 10 studies targeted and included women only (33, 42, 43, 46, 50, 52, 53, 56, 61, 68) and the remaining 29 included men and women (Table 1). Some studies recruited participants from the general public (n=13) (35, 36, 40, 51, 54–56, 59, 60, 64, 65, 67, 69), while other studies targeted people with a specific health condition as follows; diabetes or pre-diabetes (n=8) (31, 32, 34, 38, 45, 47, 61, 62), hypertension/prehypertension (n=2) (35, 58), high cardiometabolic risk or undergoing cardiac rehabilitation (n=2) (48, 57), obesity (n=2) (35, 53), mild cognitive impairment (n=1) (66), chronic kidney disease (n=1) (63), and metabolic syndrome (n=1) (49). Remaining studies targeted breast cancer survivors (n=4) (42, 43, 50, 53), post-menopausal women (n=1) (68), low income earners (n=1) (41, 43), and specific ethnic groups in the US (n=8) such as Hispanic (42), Black (39), Latinas (46), and African Americans (31, 33, 37, 44, 52) (Table 2).

**Table 2:** Overall characteristics of culinary nutrition education programs

**Author (Year) Country**	**Study design**	**Age**	**Sample size**	**Target group**	**Program activities (including post-program activities)**	**Class frequency / Program duration**	**Education topics**
Pedersen et al. (1992) United States	Pre- & post study	Range: 31+ (94.7% were 51+) Mean: not reported	43	People with diabetes	• Nutrition education with videos & a presentation of the foods included in each food exchange list with correct portion sizes • Application of Meal Planning • Subjects answered questions concerning which foods belonged in each food exchange group. • Meal & snack planning from a sample meal plan using the food exchange lists • Guidelines for eating out, alcohol use, and diet during illness • Recipes & taste testing	5 weekly classes Class duration not reported 5 weeks total.	• What Is Diabetes? components of the diabetes care plan, normal fasting and postprandial blood glucose levels. • A Guide to Meal Planning, an overview of the goals and rationale for meal planning, The dietary recommendations of the American Diabetes Association • Diabetes Exchange Lists, Application of Meal Planning, guidelines for eating out, alcohol use, and diet during illness.
Doshi et al. (1994) United States	Pre- & post study	Range: 55–88 Mean: 71.8	31	Black older adults in an urban community	• Nutrition education • Demonstration of culturally specific cooking suggestions • Physical activities lasted 30 to 40 minutes	20 bi-weekly classes. Class duration not reported 10 weeks total.	• Nutrition information on energy, fat, cholesterol, polyunsaturated and saturated fat, and sodium content of foods
Barnhart et al. (1998) United States	Pre- & post study	Range: 45–79 Mean: 60.5 ± 9.26	18	African American women	• Education session • Fruit and vegetable served every session • Discussion • Preparing a low-fat dish	3 bi-weekly sessions each 90-minutes. 8 weeks total.	• Problem solving • Understanding the role of religious or familial preferences for food • Identify goals and strategies to overcome barriers to eat fruit and vegetable • Food advertising and labelling • Setting long-term goals
Campbell et al. (1999) United States	Randomized controlled trial	Range: 18+ Mean: 53.8	2519	African American church members	• Activities targeting primarily predisposing factors (Tailored bulletins; Printed materials) Eg Gardening; Educational sessions; Cookbook and recipe tasting; Serving more fruits and vegetables at church functions • Activities targeting primarily reinforcing factors (Lay health advisors; Community coalitions; Pastor support; Grocer-vendor involvement; Church-initiated activities • Providing social support for members to advance in stages of change	Class duration and frequency: not reported Intervention consisted of multiple different activities that occurred over 20 months with no timeframe specified for each	• Modifying cooking methods • Canning and freezing produce
Yanek et al. (2001) United States	Randomized trial	Range: not reported Mean: Spiritual intervention: 53.6 ± 9 Standard intervention: 51.9 ± 9 Self-help control group: 53.9 ± 10	529 Spiritual intervention: 267 Standard intervention: 188 Self-help control group: 74	Female African American church members	• Standard intervention: 30 – 45 minutes nutrition education with taste test or cooking demonstration & 30 minutes moderate intensity aerobic activity • Spiritual intervention: same as standard intervention with the addition of: Group prayer, Health messages enriched with scripture, Aerobic and Gospel music or Praise and Worship dance, Call from lay leaders and word of mouth from other participants motivated attendance, Joy of Health: church bulletins weekly reminders and printed messages accompanied by salient scriptures, Tip sheet: regular information on healthy eating and physical activity • Self-help control group: Materials from the American Heart Association on healthy eating and physical activity and information targeted to the participants personal screening results, a gift-wrapped box with their name on it containing feedback on their personal screening results, place to list their personal goals for the year materials and the project joy an IH and YMCA educational materials and pamphlets a hotline number was available for consultation from the professional project joy health educators	Intervention: 60 to 75 minutes weekly sessions of support was available for the remining of the year. 20 weeks total	• Vegetables-Preparation • Fats/Three-Month Review • Meats • Meat Alternatives • Grains and Fiber • Dairy Foods • Salt/Sodium • Shop ‘til You Drop (the Fat) • Dining Out • Breakfast, Lunch, and Snacks • Holiday Eating • Six-Month Evaluation • The Food Guide Pyramid • Fat Counting • Portion Size • Food Labelling • Energy Balance • Fruits • Why We Eat • Vegetables -Benefits
Anderson-Loftin et al. (2002) United States	quasi-experimental study	Range: 25–77 Mean: 51	23	African American people with diabetes	• Nutrition education, cooking class, educational videos (purchasing healthy foods), Healthy snacks provided, Group discussion, Phone call follow up, Home visits	4 sessions each 1 hour at 2-week intervals. 8 weeks total	• Planning healthy meals with diabetes food pyramid • Eating at Home: group preparation of healthy African American foods. • Purchasing healthy African American foods. • Eating away from home: making healthy choices of African American foods.
Pelletier et al. (2003) United States	Pre- & post study	Range: 30 – 89 (68% were aged 60 – 79) Mean: not reported	69	Cardiac rehabilitation patients	• Education sessions involving a visual presentation using computer slides, Food samples distribution	Two 90-minute education session occurred 2 months apart	• Focused on soy, oats, sterols and nuts
Keller et al. (2006) Canada	Pre- & post study	Range: not reported Mean: 72	251	Healthy older adults of an older adults’ recreation centre	• 1st year: monthly activities including food demonstration or workshop, hand-outs and recipes, monthly display board, monthly fresh produce, individual nutrition counselling, a column in the centre’s monthly newsletter, provision of nutrition education resources for the centre’s library • 2nd year: in addition to 1st year activities: a cooking group for men and a support group for older adults with diabetes.	Monthly sessions for 2 years Session duration: not reported	• Not all topics were disclosed, but examples given were cooking for one, choosing fruits and vegetables.
Rydwik et al. (2008) Sweden	Randomized controlled trial	Range: not reported Mean: Nutrition (N) group: 83.1 ± 4.5 Training (T) group: 83.5 ± 3.7 Nutrition & Training (N+T) group: 83.1 ± 4 Control (C) group: 82.9 ± 4	96	Frail community-dwelling older adults over age 75 receiving home services in the municipality of Solna	• Nutrition program: Individual dietary counselling and five group sessions with examples of nutritionally well-balanced meals/snacks provided at each • Physical training: Warm-up (including aerobic training) Muscle-strength training Qigong (cool-down)	• Nutrition program: 1 individual dietary counselling (1 hour) and five group sessions Duration is not reported • Physical training: 2 weekly session for 40 minutes lasted 12 weeks	3 examples of 5 topics were provided: • The nutritional needs of older people, meal frequency • Cooking methods • General diet advice: to eat three main courses and 2–3 between-meal snacks including meat, fish or egg, fruit and vegetables, dairy products, and fiber, in combination with fluid every day.
Hien et al. (2008) Vietnam	Controlled trial	Range: Not reported Mean: 57.6 ± 3.0	108	Postmenopausal Vietnamese women	• Intervention group: nutrition education: - Training courses (posters, leaflets, booklet and video tape) - Disseminating education message through a loud speaker. - Monitoring intake at home - Group discussion (preparing, processing and cooking the meals based on local calcium-rich foods) • Control group: Usual diet (nutrition education provided after data collection)	Frequency and duration of training courses not specified • Daily education messages Weekly group discussions and exercises	• Osteoporosis the role of calcium intake in the disease How to identify calcium-rich foods in the locality How to prepare meals with local foods based on guided menus • Education messages: «Take calcium, rich foods every day to enhance your bone health» & «Take guided menu into your meals to reach enough calcium intake»
Wunderlich et al. (2011) United States	Pre- & post study	Range: not reported Mean: Congregate meals group: 74.45 ± 9.46 Home delivered group: 78.98 ± 9.90	355	People aged 60 years and over living in congregate, and home delivered meal locations in a northern county of New Jersey	• Congregate meal setting: education, cooking demonstrations, tips for shopping on the selected topics for approximately 1 hour with ‘question and answer’ sessions. • Home delivered meals: education materials, calls to a nutritionist for individual counselling	Congregate meal sites: At least 4 times per year, 30–40 minutes per session with 1 hour of interactive activities with additional question and answer sessions. Home delivered meals: not specified as education materials were delivered by mail or with the meal but were encouraged to call the nutritionist for free individual counselling	Intervention group education topics: • Focused on timely and common physical conditions among older adults • Hypertension and salt intake meal management for persons with diabetes
Meethien et al. (2011) Thailand	Pre- test & post- test, experimental and control group design	Experimental group Range: 60–85 Mean (SD): 67.42 (6.62) Control group Range: 60–82 Mean (SD): 66.6 (5.5)	Experimental group: 43 older adults, 43 family members Control group: 40 older adults, 40 family members	60 years and older, living in non-municipal areas of north-eastern Thailand	• Nutritional education: teaching and discussion, older adults Healthy Eating Booklet Individual counselling: problem solving activities, regarding issues they might encounter as they practiced healthy eating (i.e., barriers to adopting healthy eating; adjusting their dietary plan; setting realistic goals related to healthy eating) • Motivational interventions: demonstration of healthy food preparation and food choices, training and guidance on personal meal plan and personal goals setting, verbal reinforcement/advice to reduce emotional problems relating to eating behaviour changes, group discussions to adopt healthy eating; teaching, group discussion and individual counselling • Behavioural maintenance: Healthy eating handouts topics are: 1) Easy Steps for Healthy teaching and discussion, older adults Healthy Eating Booklet Individual counselling: problem solving activities, regarding issues they might encounter as they practiced healthy eating (i.e., barriers to adopting healthy eating; adjusting their dietary plan; setting realistic goals related to healthy eating)	Weekly 45–60 minute sessions for 3 months	• Healthy eating • Thai Food Pyramid Guide • Dietary Guidelines, and Nutrition • Facts Labels for Thai older people essential nutrients • 30 essential nutrients • Food choices and purchases • Food preparation (safety and storage) • Benefits of and barriers to healthy eating • Healthy food menus for older people in northeastern Thailand Also, Individual education regarding issues they might encounter as they practiced healthy eating (i.e., barriers to adopting healthy eating, adjusting their dietary plan, and, setting realistic goals related to healthy eating), personal meal plan and personal goals setting.
Archuleta et al. (2012) United States	Quasi-experimental pre- test & post-test study	Range: 30–85 Mean (SD): 63 ± 11	117	People with Type 2 diabetes	• Nutrition education: using the Diabetes Food Guide Pyramid in meal planning food labels, portion control identifying sources of carbohydrate heart-healthful cooking techniques • Hands on cooking: meal preparation, recipe demonstration and tasting supplemental online information	Weekly classes for 3 hours each 4 weeks total	• Meal Planning • Balancing Carbohydrates • Vegetables, Beans & Grains • Heart Healthy Cooking
Griffith et al. (2012) United States	Pre- & post study	Range: 47 – 74 Mean: 61.1 ± 3.1	8	Low- to middle-income African American breast cancer survivors	Nutritional counselling sessions: review of 3-day food records, discussion of strategies to decrease the amount of fat in the diet through goal setting, identification of barriers to dietary goals, and delivery of cognitive approaches. 2. Educational group meetings: discussion of basic nutrition principles, demonstration of low-fat recipes and a grocery store tour which focused on reading food labels and choosing heart-healthy items. 3 4.a copy of the culturally specific Women’s Intervention Nutrition Study (WINS-c) plan	Eight 45- to 60-mintue individual nutritional counselling sessions. One year total	• Unclear
Bielamowics et al. (2013) United States	Pre- & post study	Range: not reported Mean: not reported	1026	People with Type 2 diabetes	• Education session with PowerPoint presentations, videos & marketing materials. • A cookbook of Texas-style foods with tested diabetes recipes, brochures	Do Well, Be Well with Diabetes (DWBW): 9 lessons Cooking Well with Diabetes (CWWD): DWBW: 4 Sessions Frequency not reported	CWWD topics: • Healthy recipes to cut fat, sugar, and sodium and increase fiber content of foods • Carbohydrate Foods, Starch & Dessert Recipes • Making Recipes with Fat Better for You, Main Dish Recipes • Double Pleasure Side Dishes: Reducing Sodium and Increasing Fiber, Side Dish Recipes • Diabetes Reunion: Celebrating Sensibly with Diabetes, Holiday Recipes. DWBW topics: • What Is Diabetes? • Nutrition—First Step to Diabetes Management • One Diabetes Diet—No Longer the Sole Option • Managing Your Blood Glucose • Nutritional Labels • Diabetes and Exercise • For Good Measure at Home and Eating Out • Diabetes Medicines • Preventing and Managing Complications
Paes-Barreto et al. (2013) Brazil	Randomized controlled clinical trial	Range: not reported Mean: 63.4 ± 40.8	Total: 89 Normal counselling: 46 Intense counselling: 43	People with stage 3 to 5 chronic kidney disease	• Intense Counselling Group: Nutrition education: (1) an individual class (15–20 minutes) providing information on food sources of protein and sodium, reasons for reducing the intake of such food items, and the potential benefits of this therapy (2) a hands-on session on protein-rich food by using food models and household measuring utensils (3) an education folder including recipes (4) a hands-on session using test tubes with the amount of salt content in portions of some foods. • Dietary counselling: participants were prescribed low-protein, low-sodium diet based on Kidney Disease Outcomes Quality Initiative Nutrition Guidelines • Dietary Counselling Group only: participants were prescribed low-protein, low-sodium diet based on kidney recommendations from the National Kidney	4 sessions Intense counselling: 15–20 minutes Duration of other groups not reported 4–7 months total.	• Food sources of protein and sodium • Reasons for reducing the intake of such food items • Potential benefits of this therapy
Archuleta et al. (2012) United States	Quasi-experimental pre- test & post-test study	Range: 30–85 Mean (SD): 63 ± 11	117	People with Type 2 diabetes	• Nutrition education: using the Diabetes Food Guide Pyramid in meal planning food labels, portion control identifying sources of carbohydrate heart-healthful cooking techniques • Hands on cooking: meal preparation, recipe demonstration and tasting supplemental online information	Weekly classes for 3 hours each 4 weeks total	• Meal Planning • Balancing Carbohydrates • Vegetables, Beans & Grains • Heart Healthy Cooking
Griffith et al. (2012) United States	Pre- & post study	Range: 47 – 74 Mean: 61.1 ± 3.1	8	Low- to middle-income African American breast cancer survivors	Nutritional counselling sessions: review of 3-day food records, discussion of strategies to decrease the amount of fat in the diet through goal setting, identification of barriers to dietary goals, and delivery of cognitive approaches. 2. Educational group meetings: discussion of basic nutrition principles, demonstration of low-fat recipes and a grocery store tour which focused on reading food labels and choosing heart-healthy items. 3 4.a copy of the culturally specific Women’s Intervention Nutrition Study (WINS-c) plan	Eight 45- to 60-mintue individual nutritional counselling sessions. One year total	• Unclear
Bielamowics et al. (2013) United States	Pre- & post study	Range: not reported Mean: not reported	1026	People with Type 2 diabetes	• Education session with PowerPoint presentations, videos & marketing materials. • A cookbook of Texas-style foods with tested diabetes recipes, brochures	Do Well, Be Well with Diabetes (DWBW): 9 lessons Cooking Well with Diabetes (CWWD): DWBW: 4 Sessions Frequency not reported	CWWD topics: • Healthy recipes to cut fat, sugar, and sodium and increase fiber content of foods • Carbohydrate Foods, Starch & Dessert Recipes • Making Recipes with Fat Better for You, Main Dish Recipes • Double Pleasure Side Dishes: Reducing Sodium and Increasing Fiber, Side Dish Recipes • Diabetes Reunion: Celebrating Sensibly with Diabetes, Holiday Recipes. DWBW topics: • What Is Diabetes? • Nutrition—First Step to Diabetes Management • One Diabetes Diet—No Longer the Sole Option • Managing Your Blood Glucose • Nutritional Labels • Diabetes and Exercise • For Good Measure at Home and Eating Out • Diabetes Medicines • Preventing and Managing Complications
Paes-Barreto et al. (2013) Brazil	Randomized controlled clinical trial	Range: not reported Mean: 63.4 ± 40.8	Total: 89 Normal counselling: 46 Intense counselling: 43	People with stage 3 to 5 chronic kidney disease	• Intense Counselling Group: Nutrition education: (1) an individual class (15–20 minutes) providing information on food sources of protein and sodium, reasons for reducing the intake of such food items, and the potential benefits of this therapy (2) a hands-on session on protein-rich food by using food models and household measuring utensils (3) an education folder including recipes (4) a hands-on session using test tubes with the amount of salt content in portions of some foods. • Dietary counselling: participants were prescribed low-protein, low-sodium diet based on Kidney Disease Outcomes Quality Initiative Nutrition Guidelines • Dietary Counselling Group only: participants were prescribed low-protein, low-sodium diet based on kidney recommendations from the National Kidney	4 sessions Intense counselling: 15–20 minutes Duration of other groups not reported. 4–7 months total.	• Food sources of protein and sodium • Reasons for reducing the intake of such food items • Potential benefits of this therapy
Johari et al. (2014) Malaysia	Randomized controlled trial	Range: 60 – 72 Mean: 64.7 ± 3.8 Intervention: 66.9 ± 4.2 Control: 63.5 ± 2.9	35	Urban older individuals with Mild Cognitive Impairment in Kuala Lumpur, Malaysia.	• Activities on healthy dietary intake: consumption of fish, vegetables, and fruit, diet counselling, a food quiz, demonstration of healthy food preparation, aerobic exercise, a crossword puzzle, and board games. The intervention activities based on the booklet content consisted of group activities on healthy dietary instruction that emphasized consumption of fish, vegetables, and fruit, diet counselling, a food quiz, demonstration of healthy food preparation, aerobic exercise, a crossword puzzle, and board games.	Intervention group: Monthly nutrition and lifestyle education session for 12 months Control group: 12 months of supplementation only	• Lifestyle education sessions based on a booklet entitled «7 Guides to Enhance older adults Memory», comprising guidelines about eating more fish, eating more foods rich in folic acid, eating more fruits and vegetables, exercising regularly, engaging in activities to stimulate memory, stop smoking and consumption of alcohol, and maintaining a cheerful and positive attitude
Chung et al. (2014) China	Experimental pre-post study	Range: 59–95 Mean: 74.4 ± 7.8	60	People aged 55+, who lived at home alone or in a couple on a subsidized housing estate.	Group A: A 1-day free food sample each week for 3 weeks Group B: Three 1-day free food samples each week for 3 weeks. All took part in a 3-week food education program that included three seminars on nutrition with 1-day menu recipes with video demonstration of the cooking steps with free ingredients sample.	Once a week, for 3 weeks in each district	• Nutrients classification & function • Healthy food choices • Food labelling
Francis et al. (2014) United States	Prospective mixed-methods study	Range: 55–89 Mean: 72.6	60	Congregate Meal Participants enrolees included a mixture of both past and new Chef Charles (CC) participants.	• Intervention group (revised theory-based program): The sessions, consisted of a monthly 30-minute newsletter-based discussion a 4-page newsletter focusing on health and nutrition in addition to a taste-testing activity. Instructor guide: discussion about smarter goal planning, Newsletter:, Smarter Planning section (goal setting, identifying barriers, strategies to overcome barrier), focused on main topic each edition, easy to understand information presented in bullet point or tables, photos, resources list, recipes. Taste testing • Control group (traditional CC program): Instructor guide: discussed all newsletter articles, didactic education approach Newsletter: focusing on health and nutrition-related topics including monthly varied template, most information presented in paragraph form, clipart, different resources, recipes	Monthly 30-minute newsletter-based discussion course for 6 months	Intervention group • MyPlate overview (main topic: Vitamin B12) • MyPlate on budget (main topic:) • Eating healthy on a budget • MyPlate and potassium (main topic) • MyPlate and diary (main topic: bone health) • MyPlate and fiber (main topic) • MyPlate and snacking (main topic) • Physical activity • Safe food handling practices • Food security Control group (traditional CC program): • MyPlate for older adults • Meat and poultry labelling, vitamin B12 • Cost of healthy food • Potassium-rich foods, Vitamin D • Trans fat labelling • Osteoporosis • Fiber and breast cancer • Physical activity • Safe food handling practices • Food security
Monlezun et al. (2015) United States	Randomized controlled trial	Range: not reported Mean: Intervention group: 62 Control group: not reported	27	Patients with type 2 diabetes	• Intervention group: Hands-on cooking and nutrition classes. Each two-hour cooking class consisted of 30 min of didactic lessons and 90 min of cooking time • Control group: received the standard of nutrition education, RD-led MNT, consisting of a one-time RD counselling visit with a referral opportunity to an American Diabetes Association-certified diabetes education class	Two-hour cooking class including of 30 min of didactic lessons and 90 min of cooking time	• Six-module cooking and nutrition curriculum on the MD for culture-specific kitchens across different socioeconomic levels
Kennedy et al. (2015) United States	Pre- & post study	Range: 26–75 Mean: 54	Total: 37 Financial counselling group: 18 Lifestyle group: 19	African American people	• Financial counselling group: education sessions on budgeting finances, balancing payload, how to avoid repossessions and bankruptcy, individual counselling sessions, and special guest lectures. • lifestyle group: cooking demonstrations and techniques to increase physical activity, monthly visit to the program site in which the research dietitian reviewed with participants the lesson plan,and provided feedback and guidance based on current recommendations to maintain and/or prevent weight gain. All participants received T-shirts, pedometers, duffle bags, healthy snacks and meals	12 monthly classes with each class lasted 1.5-hour	Only examples of the topics reported: • Special guest lectures consisted of topics on entrepreneurship opportunities, banking, real estate, long-term disability, and living wills. Examples of the lifestyle intervention lesson plans were: • Essentials for Better Health, Portion Control, and Move those Muscles.
Menezes et al. (2015) Brazil	Randomized controlled trial	Range: not reported Mean: 57.9 ± 11.7	71	Women with diabetes	• control group: Exercises, nutrition education • Intervention group: in addition to control group activities, intervention group were divided as 1) pre-action group (10 workshops) 2)action group (10 workshops), each intervention group (pre-action and action) received a series of workshops	Three sessions weekly, one hour each session. Six months total.	• Not described
Otilingam et al. (2015) United States	Randomized controlled trial	Range: 48 – 84 Heart & brain mean: 57.1 ± 8.1 Heart mean: 60.1 ± 8 Waiting list 1 mean: 61.1 ± 10 Waiting list 2 mean: 58.1 ± 9.4	Heart & brain: 32 Heart: 33 Waiting list 1: 17 Waiting list 2: 18	Latinas	Nutrition education, cooking demonstrations and tasting, in-class exercise, fotonovelas, experience sharing, and game show formats. Vivid photographs and other visual aids are prominently featured to circumvent potential concerns of low reading literacy. The program incorporates three behaviour change principles: reducing dietary fat barriers, building dietary fat self-efficacy, and providing cues to action.	Two two-hour workshops. Two weeks total	Workshop 1: • Fat versus muscle • Benefits and barriers • The chain game • Types of fats • The colours of milk bottle cap home activity • Heart disease discussion • The brain connection • Achieve your goals by taking small steps • Local food directory • Buy healthy foods on a budget • Supermarket map • Buy for taste, not for fat • Buying snacks Workshop 2: • Food pyramid loteria • Reading food labels • Cook with less fat • Cooking class • What to cook • Portion size • Serving size • Balanced plate • Eating during holidays and celebrations • Eating out
Irwan et al. (2016) Indonesia	Randomized controlled trial	Range: not reported Mean: 66.5 ± 6.1	45	Participants with hypertension/ pre-hypertension	Educational training and maintenance meetings	Two-day educational training in a week with 90 minutes per session, followed by 10 minutes break each 90-minute maintenance meeting a month after educational training. Four months total.	• Effects of high salt intake on blood pressure. • Benefits of reducing salt intake. • Ideal daily salt intake. • Substitutes for salt to maintain appetite. • List of foods to avoid/reduce. • Challenges and solutions of a low-salt diet. • Cooking and testing low-salt menus. • Determining samples of daily low-salt menus and setting individual targets. • Sharing Benefits of salt reduction. • Overcoming Challenges of low salt intake. • Tips for Overcoming challenges. • Resetting targets.
Brewer et al. (2016) United States	Quasi-experimental pilot study	Range: 62–93 Mean: Control: 77.6 ± 8.2 Intervention: 74.1 ± 8.4	35	Community-dwelling, non-institutionalized older adults aged 60+ that attended their local older adults centre.	• Intervention group: five 15-min lessons focusing on fruit and vegetable consumption, phytochemical guide, phytochemical health information cards, recipe cards, sample of prepared recipes, meal consumption (lunch) • Control group: phytochemical guide, phytochemical health information cards, recipe cards, meal consumption (lunch)	A series of five, 15-minute fruit and vegetable-themed nutrition education lessons and educational tools. Total 4 months	Lessons topics: • Basics of phytochemicals and their health benefits • Serving sizes of fruits and vegetables • Shopping techniques and tips to overcome common barriers to eat fruit and vegetables. • Tips to overcome common barriers typically perceived by older adults in regards to incorporating a variety of fruits and vegetables into their daily meals.
Power et al. (2016) Ireland	Parallel randomised controlled trial	Control Range: 60–89 Mean: 74.4 ± 7.61 Treatment Range: 60–91 Mean: 75.3 ± 7.82	100	Adults aged over 60 years and living alone	• Intervention group: A peer volunteer visited the individuals once weekly to prepare and share a meal with them. a guidebook including nutritional and culinary information and tips as well as recipes • Control: received the guidebook containing recipes as well as nutritional and culinary information and advice, but no visitor	One weekly training for 90 minutes/session. Total 8 weeks.	Guidebook including nutritional and culinary information and tips as well as recipes designed to be quick and cost-effective.
Greenlee et al. (2016) United States	Randomized controlled trial	Total: not reported Range: not reported Mean: 56.6 ± 9.7 Intervention: Range: 40–78 Mean: 55.1 ± 9.1 Control: Range: 36 – 81 Mean: 58 ± 10.1	70 Intervention: 34 Control: 36	Hispanic women with stage O-III breast cancer	• Control: 22-page Spanish-language booklet on healthy eating for breast cancer survivor, information about Cook For Your Life. Intervention: The 9 sessions included 4 roundtables, 3 hands-on cooking classes, and 2 food shopping field trips, copy of 22-page Spanish-language booklet on healthy eating for breast cancer survivor. Nutrition roundtables (2-hour): information about the potential benefits of dietary change, and improve health literacy, interactive presentations and discussions Cooking classes (3.5 hours): hands-on cooking. Participants prepared and then shared the meal together Food shopping field trips (1.2 hours): visiting a supermarket a 1-hour discussion to review troubleshoot barriers to change	9-session 1.5 to 3.5 hours each Total 12 weeks	• Self-assessment of intake compared to dietary cancer prevention guidelines to increase concern and the perceived benefits of the targeted behaviours (or pros of change). • Skills and strategies needed to increase self-efficacy to implement change to eat a healthy diet, and skills in solving problems involving food and family, meal budgeting and meal planning to maintain change for the long term.
Wallace et al. (2016) Australia	Pre-& post study	Range: 31+ (81% were 61+) Mean: not reported	72	Healthy, independently-living, community dwelling individuals who had not been diagnosed with dementia but who had an interest in the subject.	Nutrition education, cooking, meal preparation and meal sharing.	4 sessions (3 hours each) once a week	• Nutritional aspects of vascular and neurocognitive health • Information on dietary improvements to reduce risk factors for non-communicable diseases • Ingredient and recipe selection • Planning and preparation of a two-course meal and meal sharing A major recurring theme throughout the intervention was the importance of food variety through the intake of fruits and vegetables, herbs and spices to promote healthy eating patterns and reduce NCD risk factors.
Chen et al. (2017) Taiwan	Quasi experimental two-group study	Range: 65–91 Mean: 72.78 ± 4.92	120	Living in rural and urban communities of Taiwan.	• Intervention group: received Dietary Self-Management Programme (DSMP) which includes a booklet, education, food samples The program designed to improve nutrition status by teaching skills of healthy eating awareness, helping to plan weekly actions, giving feedback on progress, peer storytelling, modelling healthy nutritional behaviours and problem-solving methods, reinterpreting physiological symptoms, using home visits and phone calls, and encouraging family support • Control group: received routine care, regular check-ups with physicians at their public health centre	Twelve weeks total. Class frequency not reported	• Healthy eating for community-dwelling older adults and focused on self-management as a strategy for managing their salt-, fluid-, fat- and cholesterol-intake behaviours.
Friedrich et al. (2017) Poland	Pre- & post study	Range: Not reported Mean: 69.9 ± 6.3	37	Women aged 60 to 85 that were students of Third Age University in Szczecin.	Lecture and workshop	Weekly classes lasting 90 minutes each (lectures and workshops). Four months total.	Lecture: • Basic information digestive system functions • Changes resulting from aging and dietary recommendations for the older population • Nutrient sources and requirements and their physiological role • Vitamins, micro- and microelements • The role of water in the digestive system general water balance in the body • Human physiology • Nutritional physiology • Dietetics • Pathophysiology of the older adults Workshops: • meal composition and • preparation, reading food labels, choosing the products with the right glycaemic index, etc.
MacNab et al. (2017) United States	Pre- & post study	Range: 60+ Mean: not reported	157	Community-residing older adults	• Education sessions: participant—instructor interaction small group discussion hands-on activities taste-testing educational resources: printed PowerPoint slides, worksheets, information handouts, recipes Sites provided both PowerPoint presentation style and non-PowerPoint presentation style sessions	Three sessions, one hour per week	«Is It Whole Grain?» sessions: • Breakfast • Lunch • Dinner • Snacks
Gans et al. (2018) United States	Cluster randomized controlled trial	Range: 18 + Mean: Total: 53.7 Intervention: 53.5 Control: 53.9	1597 Intervention: 837 Control: 760	People with low income	Discount, fresh F&V markets called ‘Fresh to You’ (FTY), a multicomponent, educational intervention, chef-run cooking demonstrations/taste-testing, a binder that included an overview of the intervention, the first month’s newsletter, three educational DVDs, 48 recipe cards and three-hole binder sleeves to store the remaining newsletters they would receive over the course of the intervention.	Two 6-week educational/motivational campaigns (duration of each session is not clear). Total 1 year	• The first campaign (‘Just Add 2’) began soon after the baseline surveys were completed and was designed to increase participants’ daily F&V consumption by two servings. • The second campaign (‘Color Your Plate’) focused on increasing the variety of F&V that participants ate
Grimaldi et al. (2018) Italy	Pre- & post study	Range: not reported Mean: Intervention: 59 ± 10 Control: 56 ± 11	106 intervention: 71 control: 35	People with high cardiometabolic risk	• Intensive intervention: Nutrition education, free-of-charge daily supply of two main meals, one meal was served at the Restaurant, and the other one was delivered to participants’ home, once a week, participants assist the chef in meal preparation and learn the recipes and the cooking methods once a week. • Conventional intervention: An individual education session with the nutritionist, a grid of recommended food choices, reinforcements of nutritional messages by the GP on a monthly basis during the 3-month intervention. Dietary prescription to the control group was based on the same nutritional recommendations followed for the meal preparation of the intervention group	Three-month intervention consisting of twelve weekly group meetings. Nine-month follow-up.	• Healthy lifestyle • Eating behaviour
Powell et al. (2018) United States	Pre- & post study	Range: 38 – 65 Mean: 53.3 ± 7.2	26	People with metabolic syndrome	Physical activity (30 minutes) Meal preparation (30 minutes) Sharing the prepared meal together with « Dinner Conversation” that focused on new content presented via demonstrations, feedback, successes, challenges, problem-solving and goal setting (60 minutes). Participants received pedometers to self-monitor their steps	A 6-month intensive phase followed by a 24-month, participant-led maintenance phase. a weekly 2-hour session (30 mins physical activity, 30mins meal preparation and 60 mins to enjoy the meal and dinner conversation) in the first 3 months and biweekly in the second 3 months basis	“Dinner conversation” focused on new content presented demonstrations, feedback, successes, challenges, problem-solving, goal setting, overcoming barriers and challenges faced
Black et al. (2019) United States	Pre- & post study	Range: not reported Mean: Single-Class Format: 62.5 ± 11 Series-Class Format: 61.6 ± 11.8	2158	Veterans Health Administration (VHA)-enrolled veterans Targeted subpopulations: obese and hypertensive veterans	Education on nutrition knowledge Practical skills such as kitchen setup, cooking, Meal experience, Grocery shopping, Label reading, Meal planning, Food budgeting	Class frequency varied across sites, with some locations only able to implement single-class offerings Sites offered “single-session” (37%) or two- to 12-session “series” (63%) formats.	Education about nutrition observation or practicing healthy cooking techniques producing and taste-testing dishes
Schneeberger et al. (2019) United States	Pre- & post study	Range: 41–73 Mean: 55.9	21	Breast cancer patients who have completed treatment	Nutrition education, culinary medicine including observe three meal preparation and taste the demonstrated dishes, practice of stress relief techniques, such as breathing exercises, guided imagery, meditation, as well as mindful sitting and eating, physical activity, practice of yoga poses and breathing exercises, a set of self-care practices	Seven 2-h classes held fortnightly. Total 14 weeks.	• How to read nutrition labels • Focus on eating more servings of unrefined, whole plant foods and less sugar, red and processed meats • Shop for healthy foods impact of unmanaged stress on health and focuses on providing patients with techniques to elicit the relaxation response
Zuniga et al. (2019) United States	Randomized controlled trial	Range: not reported Mean: Total: 57 ± 9.3 Intervention: 55.3 ± 10.3 Control: 58.4 ± 8.2	125 intervention: 60 control: 65	Early-stage, breast cancer survivors with obesity, overweight.	• Intervention: monthly nutrition and cooking workshops, Motivational Interviewing telephone calls, and individualized newsletters based on change readiness Each nutrition workshop consisted of a didactic portion with PowerPoint slides, followed by a cooking demonstration with a chef skilled in AI food preparation, a tasting, and interactive discussion among participants, study investigators, and staff. Participants received paper copies of lecture slides, a description of the properties and benefit of featured AI foods, and recipes and supply lists for the food demonstration. Workshop attendance was tracked. Participants who missed a workshop session were contacted by a patient navigator and provided with electronic copies of all materials. • Control: monthly informational brochure, telephone calls s and no navigational services	6 monthly workshops (duration not reported)	• Relationship between cancer, inflammation, and diet • Become familiar with components of the AI diet • Understand how AI foods can prevent cancer • Understand American Cancer Society recommendations to prevent cancer recurrence and spread Learn how to reduce risk of recurrence or spread by using key food groups in one’s own kitchen • Exploring nature’s anti-cancer cuisine • Understand AI foods as chemoprevention-which foods when consumed regularly have an anti-inflammatory effect • Understand the value of anti-oxidants and how they fit into the AI diet, which foods when consumed daily have an anti-oxidant effect • Learn how to reduce risk of recurrence or spread by consuming more anti-oxidant—rich foods • Understand regulations governing supplements and which products have been scientifically proven to work • Understand that supplements may be unnecessary if the diet provides adequate nutrients • Learn about Vitamin D and calcium, and why they are recommended as dietary supplements • Understand how the objectives of workshops 1–5 work together to impact recurrence risk and prevention of future cancers • Learn how the right type of chocolate can be beneficial in an anti-inflammatory diet
Dexter et al. (2019) United States	Pre- & post study	Range: 41 – 71+ (69.3% were 60+)	75 face to face group: 65 Clinical Video Telehealth (CVT): 10	Veterans with prediabetes or diabetes	Cooking and nutrition education classes to provide nutrition education, cooking instructions, budget-friendly menu building, and food safety and sanitation guidelines.	5 classes over 12 weeks (duration is not reported)	• Carbohydrate counting • Safety and sanitation • Meal planning • Creating budget-friendly recipes
Muchiri et al. (2021) South Africa	Randomized controlled trial	Range: not reported Mean: 57.2 ± 6.6	48	People with type 2 diabetes mellitus (T2DM)	• Intervention group: nutrition education, 15-minute individual session, workbook, education materials, 5–10 minutes of group exercise, activities such as testing food, healthy snacks & plan a menu, bi-monthly follow-up sessions • Control group: 2.5-hour group education only	7 sessions, held monthly duration not reported	• What is diabetes mellitus & how is it treated? (Issue of education materials) • Balancing the meals for health: Dietary guidelines • Balancing the portions • Improving intake of vegetables, fruits and legumes Planning meals on a tight budget • Preventing complications and improving quality of life • Summary and evaluation

### Program curriculum

Nutrition education content varied based on program aims and target population (Table 2). Some programs focused on disease management and provided specific dietary instructions to better manage diseases. Examples of this included reduction of salt intake in people with hypertension, management of low protein intake in people with chronic kidney diseases and increasing of calcium intake in postmenopausal women. Overall, the majority of programs (n=36) were reported as promoting healthy eating as per the relevant national health guidelines. With regards to specific diets, the Mediterranean diet was promoted in three different programs including one in a Mediterranean population (57), one for people with diabetes (57) and one for breast cancer survivors (53). Some programs reported the use of printed materials (n=18) (34, 36, 37, 40–43, 51–54, 59, 60, 63, 64, 66–68). Only a small number (n=6) used videos in the programs’ activities (31, 34, 41, 47, 55, 68). All programs were delivered face-to-face and only one program used both face-to-face and an online-telehealth approach (38).

### Integrative cooking component

The integrative cooking component highly varied across the studies. However, the most common activities were taste testing (n=19) (31–33, 36, 37, 40, 41, 44, 46–48, 52–55, 59, 62, 63, 69), viewing a cooking demonstration (n=13) (32, 39, 41, 43, 46, 50–53, 57, 60, 66, 67), the act of cooking (n= 11) (31, 32, 35, 42, 44, 45, 49, 57, 64, 65, 68), and sharing the meal or eating (n=5) (36, 42, 49, 64, 65). Few programs reported using videos (n=6) (31, 34, 41, 47, 55, 68) or visual aids like PowerPoint slides or posters (n=7) (34, 46, 48, 53, 59, 68) to deliver information. Only two programs had an online component to provide additional information for participants (32, 35).

### Theoretical framework

Of the 39 included articles, 18 studies used a behavioural theory to guide the development and implementation of program activities (31, 32, 34, 37, 40–42, 46, 49, 52, 54, 58–62, 64, 65). Theories underpinning programs varied, with some combining elements from two theories. The most commonly used theory was Social Cognitive Theory (n=8) followed by Self-Efficacy Theory (n=3) and Social Marketing Theory (n=2) (Table S1).

### Stakeholder engagement

Ten programs reported meeting with stakeholders, who were primarily end-users such as older adults, to determine their needs before designing the program (31, 33, 40, 41, 43, 52, 55, 59, 62, 67). 4 of 10 programs were targeting older adults (Table 1). The most common method of engagement was via focus groups.

### Programs outcomes

#### Dietary outcomes

There were five categories of reported dietary outcomes; (i) Dietary behaviours (n=12) (31, 34, 35, 38, 41, 45, 46, 48, 51, 57, 60, 65), (ii) Overall dietary intake (n=12) (32, 38–40, 42, 44, 47, 52, 56, 61, 62, 66), (iii) Fruit and vegetable intake (n=6) (33, 36, 37, 41, 43, 50), (iv) Malnutrition (n=6) (40, 51, 54, 55, 64, 67) and (v) other specific nutrient/food based outcome such as protein (63), calcium (68), salt (58), whole grain (59), adherence to the Mediterranean diet (53), and functional food (48). Malnutrition was measured only in the programs that specifically targeted older adults (n=6). The most common assessment tools were 3-day food records (n=5) and 24-hour food recall (n=4). Most programs (n=32) showed improvement in the dietary outcomes assessed, however 6 reported no change.

#### Non-dietary outcomes

Non-dietary outcomes varied widely and included behavioural measures such as knowledge, attitudes, beliefs, practice, self-efficacy and physiological measures such as lipid profile, blood pressure, physical activity, and cognitive status. The most common non-dietary assessments used included anthropometric measures (n=15), knowledge (n=12) for example knowledge about whole grain and diabetes treatment regimen, and blood markers, with the most common biomarker collected being Triglycerides (n=9).

Further details on study outcomes are presented in Table S2.

## Discussion

The aim of this review was to synthesise existing published literature in relation to culinary nutrition education programs that are aimed at improving the health and wellbeing of community-dwelling older adults. A total of 39 studies were identified and included in the review.

Of the included programs, 15 did not specifically target older adults and there were large variations in the reported culinary nutrition education programs. Examples of variations included the number, frequency and duration of sessions, session topics, group sizes, interactive format (i.e. viewing recipe demonstration or tasting food), providing companionship and sharing meals. Most programs measured outcomes pre-and post-program. Longer term outcomes at 2- or 3-years post-program were only reported by few studies (49, 52, 67).

Programs based on a behaviour change theory showed positive changes in dietary outcomes. This aligns with previous findings in the literature that showed favourable behaviour change using a theoretical framework to design health promotion programs (21, 25). Furthermore, findings showed that some study teams engaged with end-users as co-researchers, either through the conducting of focus groups with end users prior the program, and/or conducting of continuous evaluation during program to identify and ensure programs met their population needs. For example, the Evergreen Action program had an older adults’ advisory group, who reported that in their particular population, older men faced difficulties with cooking. In response to feedback, the team designed additional workshops focused on men called “Men can Cook” (67). These workshops helped participants to improve their confidence in cooking and finding healthy food alternatives (70). Engaging stakeholders and end-users in the design of programs can play a significant role in its effectiveness. Culinary nutrition education and cooking classes are more likely to succeed if they first identify participant needs and involve them in the design process (25, 71), as this ensures the program is relevant and beneficial for the stakeholders (72).

Programs tended to focus on country-specific dietary guidelines for older adults. Despite the Mediterranean diet being promoted for specific disease populations, there were no interventions that promoted one specific dietary approach over another for the general older population. Surprisingly, most programs were face-to-face only and did not offer online components or delivery except three programs (32, 35, 38). The program that used telehealth classes in some sites showed that there were no significant differences in outcomes between the face-to-face and telehealth delivery modes. Online and telehealth programs can be effective in older adults to promote health and can be beneficial for delivery with impacts such as covid (73–75). Given many programs encouraged eating a meal together, this may be why programs tended to be face-to-face. With telehealth or online technologies consuming a meal may still be achieved however has not yet been tested and explored as would also require use of technology and owning of devices which may be more limited in older adults than when compared with younger population groups (76). However, studies on older adults during COVID have shown that older adults are online, and they found online programs helpful (75). Online culinary nutrition programs will show recipes demonstrations and reach older adults who would not usually go to these programs due to some limitations and could include a virtual socialising group. Future programs might consider remote delivery or going online after the spread of global pandemic COVID-19. Reported studies were conducted before COVID-19.

Although visual aids (e.g. videos, handouts) are widely available and commonly used tools to deliver cooking demonstrations, few studies reported using them or reporting their use. Whole Grain nutrition education program provided some evidence on the benefit of using visuals to maintain active engagement with content. This program had two delivery modes, PowerPoints slides (sites=13) and non-PowerPoint slides (sites=12), reported that while all participants showed improvement in knowledge acquisition, participants who joined PowerPoint-based classes had significantly higher knowledge scores than participants in the non-PowerPoint-based classes (59). The decision to use the PowerPoint slides was based on older adults’ preference from a needs assessment conducted before designing the program. All participants from both delivery modes showed a significant increase in frequencies of eating whole-grain foods. Collectively visual aids help to reinforce health information especially in people with low literacy, leading to better comprehension, adherence, and outcomes (77).

This review identified the absence of online programs that offer flexibility compared to face-to-face programs. Reported face-to-face programs were limited by class size, specific geographic location, time, and inclusion criteria such as ethnicity or church membership. These limitations may have existed for a variety of reasons such as limited resources, limited budget, and cost of foods. Online programs, on the other hand, reach a larger number of participants with lower cost. They can provide an opportunity for participation for vulnerable older adults and others who might be restricted from attending face-to-face programs due to location, transportation restrictions or health status. Online programs also can provide unlimited access to evidence-based content giving older adults a reliable and practical source of information. Access to evidence-based content is especially important as research has shown that many older adults were confused by changing and mixed messages in public nutrition campaigns which made them question the credibility of food messages (78). Our findings show a need to develop online culinary nutrition education programs targeting older adults considering the COVID-19 pandemic. Such a program will provide a nutrition knowledge base for preparing meals at home and maintaining healthy eating patterns. This scoping review can inform researchers and identify implications for practice to maintain health for older adults.

Although this study provided an in-depth review of the literature on culinary nutrition education programs for older adults, this study has some limitations. It only included papers that were published in peer-reviewed journals, and was limited to English language, excluding any articles that may be relevant that do not meet these specific criteria. As this was a scoping review, a quality assessment of included papers was not conducted, so no evaluation on the quality of the included articles can be made. Information about the programs was inconsistently reported; this was a particular issue in regard to the structure of education topics included in the programs.

Strengths of this review include an extensive search conducted in five electronic bibliographic databases, ensuring a broad search of the literature. Search terms included were comprehensive, and there was no time limit applied capturing programs available in the literature.

## Conclusion

Culinary nutrition education programs may provide an optimal environment to improve the dietary habits and health of older adults. Despite this, very few programs have intentionally designed for older adults. This review provides a summary to inform researchers and policy makers on current culinary nutrition programs for older adults. Further research assessing the quality and effectiveness of cooking and nutrition programs is needed. In the light of the COVID-19 pandemic, it appears that very few online-only programs exist, indicating a need for new online programs to be designed for older adults.

## Electronic supplementary material

Appendix - Additional files
