# Supplementary material for: Culinary Nutrition Education Programs in Community-Dwelling Older Adults: A Scoping Review
Source: J Nutr Health Aging. 2023 Jan 24;27(2):142–58. doi: 10.1007/s12603-022-1876-7 (PMC9872757; doi:10.1007/s12603-022-1876-7)
Supplement: Supplementary file 1 — Appendix - Additional files [file 12603_2022_1876_MOESM1_ESM.docx]

**Appendix - Additional files:**

## Summary of dietary outcomes and behavior theory

**Table S1:** Summary of dietary outcomes and behaviour theory

| **Author** | **Dietary Outcomes** | | | | | **Dietary assessment tools** | **Behaviour theory** |
| --- | --- | --- | --- | --- | --- | --- | --- |
|  | **Malnutrition** | **Dietary behaviour** | **Food intake** | **Fruit & Vegetables** | **Other** |  |  |
| Pedersen et al. |  |  | 🗸 |  |  | 4-day food record | - |
| Doshi et al. |  |  | 🗸 |  |  | 24-hour recall | - |
| Barnhart et al. |  |  |  | 🗸 |  | Food Frequency Questionnaire | - |
| Campbell et al. |  |  |  | 🗸 |  | 15-item Food Frequency Questionnaires | Concepts from the stages-of-change transtheoretical model, social cognitive theory, and social support models.  Concepts from these models organized into a framework of activities using the "PRECEDE-PROCEED" model. |
| Yanek et al. |  |  | 🗸 |  |  | Block Food Questionnaire (food frequency instrument) | Social Learning Theory |
| Anderson-Loftin et al. |  | 🗸 |  |  |  | Food Habits Questionnaire | Conceptual Model of Nursing Case Management for Rural Setting |
| Pelletier et al. |  | 🗸 |  |  | Functional food consumption frequency | Survey | - |
| Keller et al. | 🗸 |  |  |  |  | SCREEN* | - |
| Rydwik et al. |  |  |  |  | Energy intake | 4-day food record | - |
| Hien et al. |  |  |  |  | Calcium | Semi-Quantitative Food Frequency Questionnaire | - |
| Wunderlich et al. | 🗸 | 🗸 |  |  |  | Nutrition Survey risk Screening questionnaire | - |
| Meethien et al. |  | 🗸 |  |  |  | Self-Dietary Assessment Form (SDAF) | Pender’s Health Promotion Model |
| Archuleta et al. |  |  | 🗸 |  |  | 3-day recall | Social Cognitive Theory |
| Griffith et al. |  |  |  | 🗸 | Fat | 3-day recall | - |
| Bielamowics et al |  | 🗸 |  |  |  | Questionnaire | Adult learning theory, Health Belief Model, Public Health Nursing Model, Transtheoretical model |
| Paes-Barreto et al. |  |  |  |  | Protein intake | 24-hour recall | - |
| Johari et al. |  |  | 🗸 |  |  | Face-to-face interview using a Diet History Questionnaire (DHQ) | - |
| Chung et al | 🗸 |  |  |  |  | Mini Nutritional Assessment (MNA) | - |
| Francis et al. | 🗸 |  | 🗸 |  |  | Dietary Screening Tool (DST) | Health Belief Model, Social Marketing Theory |
| Monlezun et al. |  | 🗸 |  |  |  | Dietary habits, attitudes, and competencies (DACs) survey | - |
| Kennedy et al. |  |  | 🗸 |  |  | PBRC Food Frequency Questionnaire (FFQ)* | - |
| Menezes et al. |  |  | 🗸 |  |  | 24-hour recall | Transtheoretical Model |
| Otilingam et al. |  | 🗸 |  |  |  | Fat-related diet habits questionnaire | Used three behaviour change principles: reducing dietary fat barriers, building dietary fat self-efficacy, and providing cues to action |
| Irwan et al. |  |  |  |  | Salt in food | Compact Salt Meter to measure salt in food and aKME-03 salinity checker for salt in urine | Self-care Deficit Theory, Self-efficacy Theories, Geragogy Learning Model |
| Brewer et al. |  |  |  | 🗸 | Phytochemical index score | Plate waste measurement | - |
| Power et al. | 🗸 |  |  |  | Energy intake | 24-h dietary recall  Nestle mini-nutrition assessment | Social Cognitive Theory |
| Greenlee et al. |  |  | 🗸 |  |  | 3-day recall | Social Cognitive Theory, the Stages of Change Construct Transtheoretical Model |
| Wallace et al. |  | 🗸 |  |  |  | 18-item food frequency and dietary habit questionnaire | Social Cognitive Theory |
| Chen et al. | 🗸 |  |  |  |  | 18-item MNA | Bandura’s Self-efficacy Theory |
| Friedrich et al. |  |  | 🗸 |  |  | 3-day recall | - |
| MacNab et al. |  |  |  |  | Whole Grain | DST | Social Marketing Theory |
| Gans et al. |  | 🗸 |  | 🗸 |  | National Cancer Institute’s ‘Eating at America’s Table All Day’ Screener | Social Cognitive Theory, Social ecological model |
| Grimaldi et al. |  | 🗸 |  |  |  | 7-day recall | - |
| Powell et al. |  |  |  | Vegetable including non-starchy | Sweetened beverages | All-Day version of the Fruit and Vegetable Screener | Social Cognitive Theory, Self-determination theory |
| Black et al. |  | 🗸 |  |  |  | 14-item questionnaire | - |
| Schneeberger et al. |  |  |  | 🗸 | Fiber, Fat intake | Block dietary fat screener | - |
| Zuniga et al. |  |  |  |  | Mediterranean diet adherence, spice and herb consumption | 3-day recall | - |
| Dexter et al. |  | 🗸 |  | 🗸 | Whole grain and lean protein | The Healthy Habits Questionnaire | - |
| Muchiri et al. |  |  | 🗸 | 🗸 | Starchy foods, Energy intake | Two face-to-face 24hr diet recalls | Social Cognitive Theory |

*PBRC Food Frequency Questionnaire (FFQ): Pennington Biomedical Research Center’s modified Food Frequency Questionnaire

SCREEN: Seniors in the Community: Risk evaluation for eating and nutrition survey

## Summary of non-dietary outcomes

## **Table S2:** Summary of non-dietary/ other outcomes

| **Author** | **Anthropometrics** | **Behavioural outcomes** | | | | **Blood markers** | | | | | | **Exercise** | **Uncategorised/ Other** |  |
| --- | --- | --- | --- | --- | --- | --- | --- | --- | --- | --- | --- | --- | --- | --- |
|  |  | **Knowledge** | **Attitude** | **Practice** | **self-efficacy** | **HbA1c** | **glucose** | **cholesterol** | **TGL*** | **LDL/**  **HDL*** | **Blood**  **pressure** |  |  |  |
| Pedersen et al. |  | 🗸 |  |  |  |  |  |  |  |  |  |  | - |  |
| Doshi et al. | 🗸 |  |  |  |  |  | 🗸 | 🗸 | 🗸 |  |  | 🗸 | - |  |
| Barnhart et al |  | 🗸 |  | 🗸 |  |  |  |  |  |  |  |  | - |  |
| Campbell et al. |  | 🗸 |  |  | 🗸 |  |  |  |  |  |  |  | Stage of change, availability of fruits and vegetables at church functions. |  |
| Yanek et al. | 🗸 |  |  |  |  |  |  | 🗸 | 🗸 | 🗸 | 🗸 | 🗸 | Heart rate, smoking and carbon monoxide |  |
| Anderson-Loftin et al. | 🗸 |  |  |  |  | 🗸 | 🗸 | 🗸 | 🗸 | 🗸 | 🗸 |  | Health services utilization, access & cost of care |  |
| Pelletier et al. |  | 🗸 | 🗸 |  |  |  |  |  |  |  |  |  | Social norms, health history data |  |
| Keller et al. |  | 🗸 | 🗸 |  |  |  |  |  |  |  |  |  | Participation in EAN activities |  |
| Rydwik et al. | 🗸 |  |  |  |  |  |  |  |  |  |  |  | Physical performance |  |
| Hien et al. | 🗸 |  |  |  |  |  |  |  |  |  |  |  | Serum Parathyroid hormone levels, bone mass |  |
| Wunderlich et al. |  |  |  |  |  |  |  |  |  |  |  |  | - |  |
| Meethien et al. |  |  |  |  |  |  |  |  |  |  |  |  | - |  |
| Archuleta et al. |  |  |  |  |  |  |  |  |  |  |  |  | - |  |
| Griffith et al. |  |  |  |  |  |  | 🗸 | 🗸 | 🗸 | HDL only |  |  | Insulin |  |
| Bielamowics et al |  | 🗸 |  |  |  |  |  |  |  |  |  |  | - |  |
| Paes-Barreto et al. | 🗸 |  |  |  |  |  | 🗸 |  |  |  |  |  | Creatinine, urea, phosphorus, potassium, hemoglobin, albumin, hematocrit, glomerular filtration rate (GFR) |  |
| Johari et al. |  | 🗸 | 🗸 | 🗸 |  |  |  |  |  |  |  |  | Cognitive status, serum B12, red cell folate, serum homocysteine |  |
| Chung et al |  |  |  |  |  |  |  |  |  |  |  |  | - |  |
| Francis et al. |  |  |  |  | 🗸 |  |  |  |  |  |  |  | - |  |
| Monlezun et al. | 🗸 |  |  |  |  | 🗸 |  | 🗸 | 🗸 | 🗸 | 🗸 |  | Food security, heart rate, hypoglycemic agents and insulin |  |
| Kennedy et al. | 🗸 |  |  |  |  |  |  |  |  |  | 🗸 | 🗸 | Quality of life, Health questionnaires, Household food security status |  |
| Menezes et al. | 🗸 |  |  |  |  |  |  |  |  |  |  |  | Readiness to change consumption of fats |  |
| Otilingam et al. |  |  |  |  |  |  |  |  |  |  |  |  | - |  |
| Irwan et al. | 🗸 | 🗸 | 🗸 |  | 🗸 |  |  |  |  |  | 🗸 |  | Cognitive levels, hypertension history assessment |  |
| Brewer et al. |  | 🗸 |  |  |  |  |  |  |  |  |  |  | Perceived health, Self-reported health history, Lunch quality questionnaire, Meal satisfaction |  |
| Power et al. |  |  |  |  | 🗸 |  |  |  |  |  |  |  | Social connectedness/social isolation, Health status, Food enjoyment |  |
| Greenlee et al. | 🗸 |  |  |  |  |  | 🗸 |  |  |  |  |  | - |  |
| Wallace et al. |  | 🗸 | 🗸 | 🗸 |  |  |  |  |  |  |  |  | Confidence |  |
| Chen et al. |  |  |  |  | 🗸 |  |  |  |  |  |  |  | health control beliefs |  |
| Friedrich et al. | 🗸 |  |  |  |  |  | 🗸 |  | 🗸 | 🗸 |  |  | Keys’ atherogenic score |  |
| MacNab et al. |  | 🗸 |  |  |  |  |  |  |  |  |  |  | Opinions related to whole grains, lifestyle habits |  |
| Gans et al. |  |  |  |  |  |  |  |  |  |  |  |  | - |  |
| Grimaldi et al. | 🗸 |  |  |  |  | 🗸 | 🗸 | 🗸 | 🗸 | 🗸 | 🗸 |  | - |  |
| Powell et al. |  |  |  |  |  |  |  |  |  |  |  | 🗸 | Depression, Metabolic syndrome remission, social support, energy and vitality |  |
| Black et al. |  |  |  |  |  |  |  |  |  |  |  |  | Cooking confidence, Label-reading |  |
| Schneeberger et al. | 🗸 |  |  |  |  |  |  |  |  |  |  |  | Depression, perceived stress scale, patient activation, physical and mental quality of life |  |
| Zuniga et al. |  |  |  |  |  |  |  |  |  |  |  |  | Treatment length and modality |  |
| Dexter et al. | 🗸 |  |  |  |  | 🗸 |  | 🗸 | 🗸 | 🗸 |  |  | - |  |
| Muchiri et al. |  | 🗸 |  |  | 🗸 | 🗸 |  | 🗸 | 🗸 | 🗸 | 🗸 |  | - |  |
| EAN: Evergreen Action Nutrition  TGL: triglycerides  LDL/ HDL: low-density lipoprotein/ High-density lipoprotein | | | | | | | | | | | | | | |

## Electronic search strategy for Scopus

TITLE-ABS-KEY ( cook*  OR  culinary  OR  eating  OR  dietary  OR  ( ( meal*  OR  food )  W/3  ( budget*  OR  plan*  OR  skill*  OR  knowledge  OR  education*  OR  prepar*  OR  literac*  OR  shop* ) ) )  AND  TITLE-ABS-KEY ( ( ( nutrition* )  W/5  ( intervention*  OR  program*  OR  workshop  OR  workshops  OR  knowledge  OR  education ) ) )  AND  TITLE-ABS-KEY ( ( ( aged  OR  elderly  OR  older  OR  senior )  W/3  ( male*  OR  female*  OR  men  OR  women  OR  citizen*  OR  person  OR  people  OR  population ) ) )  AND  ( LIMIT-TO ( EXACTKEYWORD ,  "Human" )  OR  LIMIT-TO ( EXACTKEYWORD ,  "Humans" ) )  AND  ( LIMIT-TO ( LANGUAGE ,  "English" ) )

## PRISMA-ScR checklist

Preferred Reporting Items for Systematic reviews and Meta-Analyses extension for Scoping Reviews (PRISMA-ScR) Checklist

| **SECTION** | **ITEM** | **PRISMA-ScR CHECKLIST ITEM** | **REPORTED ON PAGE #** |
| --- | --- | --- | --- |
| **TITLE** | | | |
| Title | 1 | Identify the report as a scoping review. | 1 |
| **ABSTRACT** | | | |
| Structured summary | 2 | Provide a structured summary that includes (as applicable): background, objectives, eligibility criteria, sources of evidence, charting methods, results, and conclusions that relate to the review questions and objectives. | 2 |
| **INTRODUCTION** | | | |
| Rationale | 3 | Describe the rationale for the review in the context of what is already known. Explain why the review questions/objectives lend themselves to a scoping review approach. | 3, 4 |
| Objectives | 4 | Provide an explicit statement of the questions and objectives being addressed with reference to their key elements (e.g., population or participants, concepts, and context) or other relevant key elements used to conceptualize the review questions and/or objectives. | 4 |
| **METHODS** | | | |
| Protocol and registration | 5 | Indicate whether a review protocol exists; state if and where it can be accessed (e.g., a Web address); and if available, provide registration information, including the registration number. | 4 |
| Eligibility criteria | 6 | Specify characteristics of the sources of evidence used as eligibility criteria (e.g., years considered, language, and publication status), and provide a rationale. | 5 |
| Information sources* | 7 | Describe all information sources in the search (e.g., databases with dates of coverage and contact with authors to identify additional sources), as well as the date the most recent search was executed. | 4, 5 |
| Search | 8 | Present the full electronic search strategy for at least 1 database, including any limits used, such that it could be repeated. | 40 |
| Selection of sources of evidence† | 9 | State the process for selecting sources of evidence (i.e., screening and eligibility) included in the scoping review. | 5 |
| Data charting process‡ | 10 | Describe the methods of charting data from the included sources of evidence (e.g., calibrated forms or forms that have been tested by the team before their use, and whether data charting was done independently or in duplicate) and any processes for obtaining and confirming data from investigators. | 5& 6 |
| Data items | 11 | List and define all variables for which data were sought and any assumptions and simplifications made. | 5 |
| Critical appraisal of individual sources of evidence§ | 12 | If done, provide a rationale for conducting a critical appraisal of included sources of evidence; describe the methods used and how this information was used in any data synthesis (if appropriate). | NA |
| Synthesis of results | 13 | Describe the methods of handling and summarizing the data that were charted. | 5 |
| **RESULTS** | | | |
| Selection of sources of evidence | 14 | Give numbers of sources of evidence screened, assessed for eligibility, and included in the review, with reasons for exclusions at each stage, ideally using a flow diagram. | 6 |
| Characteristics of sources of evidence | 15 | For each source of evidence, present characteristics for which data were charted and provide the citations. | 6, 7 & 20 |
| Critical appraisal within sources of evidence | 16 | If done, present data on critical appraisal of included sources of evidence (see item 12). | NA |
| Results of individual sources of evidence | 17 | For each included source of evidence, present the relevant data that were charted that relate to the review questions and objectives. | 20 -39 |
| Synthesis of results | 18 | Summarize and/or present the charting results as they relate to the review questions and objectives. | 5 - 8 |
| **DISCUSSION** | | | |
| Summary of evidence | 19 | Summarize the main results (including an overview of concepts, themes, and types of evidence available), link to the review questions and objectives, and consider the relevance to key groups. | 9 -11 |
| Limitations | 20 | Discuss the limitations of the scoping review process. | 10 |
| Conclusions | 21 | Provide a general interpretation of the results with respect to the review questions and objectives, as well as potential implications and/or next steps. | 11 |
| **FUNDING** | | | |
| Funding | 22 | Describe sources of funding for the included sources of evidence, as well as sources of funding for the scoping review. Describe the role of the funders of the scoping review. | 12 |

JBI = Joanna Briggs Institute; PRISMA-ScR = Preferred Reporting Items for Systematic reviews and Meta-Analyses extension for Scoping Reviews.

* Where *sources of evidence* (see second footnote) are compiled from, such as bibliographic databases, social media platforms, and Web sites.

† A more inclusive/heterogeneous term used to account for the different types of evidence or data sources (e.g., quantitative and/or qualitative research, expert opinion, and policy documents) that may be eligible in a scoping review as opposed to only studies. This is not to be confused with *information sources* (see first footnote).

‡ The frameworks by Arksey and O’Malley (6) and Levac and colleagues (7) and the JBI guidance (4, 5) refer to the process of data extraction in a scoping review as data charting*.*

§ The process of systematically examining research evidence to assess its validity, results, and relevance before using it to inform a decision. This term is used for items 12 and 19 instead of "risk of bias" (which is more applicable to systematic reviews of interventions) to include and acknowledge the various sources of evidence that may be used in a scoping review (e.g., quantitative and/or qualitative research, expert opinion, and policy document).

*From:* Tricco AC, Lillie E, Zarin W, O'Brien KK, Colquhoun H, Levac D, et al. PRISMA Extension for Scoping Reviews (PRISMAScR): Checklist and Explanation. Ann Intern Med. 2018;169:467–473. [doi: 10.7326/M18-0850](http://annals.org/aim/fullarticle/2700389/prisma-extension-scoping-reviews-prisma-scr-checklist-explanation).
